# A model of access combining triage with initial management reduced waiting time for community outpatient services: a stepped wedge cluster randomised controlled trial

**DOI:** 10.1186/s12916-018-1170-z

**Published:** 2018-10-19

**Authors:** Katherine E. Harding, Sandra G. Leggat, Jennifer J. Watts, Bridie Kent, Luke Prendergast, Michelle Kotis, Mary O’Reilly, Leila Karimi, Annie K. Lewis, David A. Snowdon, Nicholas F. Taylor

**Affiliations:** 1 Eastern Health, Level 2/5 Arnold Street, Box Hill, VIC 3128 Australia; 2 La Trobe University, Kingsbury Drive, Bundoora, VIC 3086 Australia; 3 Deakin University, 221 Burwood Highway, Burwood, VIC 3125 Australia; 4 University of Plymouth, Drake Circus, Plymouth, Devon PL4 8AA UK; 5 Victorian Department of Health and Human Services, 50 Lonsdale Street, Melbourne, VIC 3000 Australia

**Keywords:** Waiting lists, Access, Appointments and schedules, Outpatients, Community health

## Abstract

**Background:**

Long waiting times are associated with public community outpatient health services. This trial aimed to determine if a new model of care based on evidence-based strategies that improved patient flow in two small pilot trials could be used to reduce waiting time across a variety of services. The key principle of the Specific Timely Appointments for Triage (STAT) model is that patients are booked directly into protected assessment appointments and triage is combined with initial management as an alternative to a waiting list and triage system.

**Methods:**

A stepped wedge cluster randomised controlled trial was conducted between October 2015 and March 2017, involving 3116 patients at eight sites across a major Australian metropolitan health network.

**Results:**

The intervention reduced waiting time to first appointment by 33.8% (IRR = 0.663, 95% CI 0.516 to 0.852, *P* = 0.001). Median waiting time decreased from a median of 42 days (IQR 19 to 86) in the control period to a median of 24 days (IQR 13 to 48) in the intervention period. A substantial reduction in variability was also noted. The model did not impact on most secondary outcomes, including time to second appointment, likelihood of discharge by 12 weeks and number of appointments provided, but was associated with a small increase in the rate of missed appointments.

**Conclusions:**

Broad-scale implementation of a model of access and triage that combined triage with initial management and actively managed the relationship between supply and demand achieved substantial reductions in waiting time without adversely impacting on other aspects of care. The reductions in waiting time are likely to have been driven, primarily, by substantial reductions for those patients previously considered low priority.

**Trial registration:**

Australian New Zealand Clinical Trials Registry ACTRN12615001016527 registration date: 29/09/2015.

## Background

Excessive waiting times for care can be a problem for both patients and health services [1]. Access issues are often associated with emergency departments [2, 3] and surgical procedures [4, 5]. However, sub-acute and community-based services also suffer from the constant pressure of lengthy waiting lists [6–9]. Delays in access to care for these services have been associated with poorer patient outcomes [10, 11], anxiety [12], and reduced engagement with services [13, 14].

Delays in care can result in waiting lists or queues. Delays, and hence waiting lists, are the result of a disparity between demand for a service and the capacity available to meet this demand [15]. Queueing theory is the equation that defines the relationship between demand, capacity and wait time [16]. Queues or waiting lists are formed when demand is higher than capacity [16]. Shortages in markets are often corrected through a “price signal”, but this mechanism is ineffective in public health services where consumers face subsidised prices and costs to consumers do not change in response to rise in demand. On the supply side, individual providers may not be responsive to price signals due to wages paid as salary or other government regulation. Hence, alternative strategies are needed to mitigate the adverse effects of long waiting times.

One strategy is to use short-term strategies such as immediate and temporary increases in supply to “clear” a waiting list; however, this does not resolve the underlying problem and waiting lists are likely to simply recur [17]. Another common strategy is to focus attention on managing the waiting list, for example, by dedicating staff to monitoring patients on the list, setting up data systems to record waiting list data or creating complex sorting or triage systems to prioritise patients according to need. These systems can help the most urgent patients to access timely care, but often do not assist in reducing overall waiting time; conversely, they may contribute to the problem by diverting resources from clinical care to administrative processes associated with managing the waiting list [17, 18].

In contrast, promising results have been reported from strategies that address patient flow by reducing complexity in booking systems, combining triage with initial management and/or actively managing the relationship between supply and demand [18, 19]. Trials in this area have focussed primarily on emergency departments (for example by placing a senior physician at triage to commence treatment and quickly manage simple cases) [20] and primary care settings (the Advanced Access approach, for example, reduces time for pre-booked appointments, opening sufficient space for patients needing same-day or next-day consultations with their local doctor) [21]. Preliminary evidence is also available from isolated studies in single services to suggest that patient flow interventions that utilise one or more of these elements may be effective in other types of community outpatient services [22–25].

Long waits for services are a problem for community outpatient services with negative consequences for patients and service providers. Of the various strategies that have been tried to reduce waiting time, there are several elements that show promise of effectiveness but evidence is limited to small, single-site studies or from extrapolation of evidence from emergency departments and primary care settings. It is not known whether a model of care that brings these key elements together can be successful in reducing waiting time across a variety of community-based outpatient health services. This trial aimed to determine whether a model of access that combines triage with initial management and allows supply to be responsive to demand fluctuations can be used to reduce waiting time across multiple community outpatient services.

## Methods

### Design

**Fig. 1:**
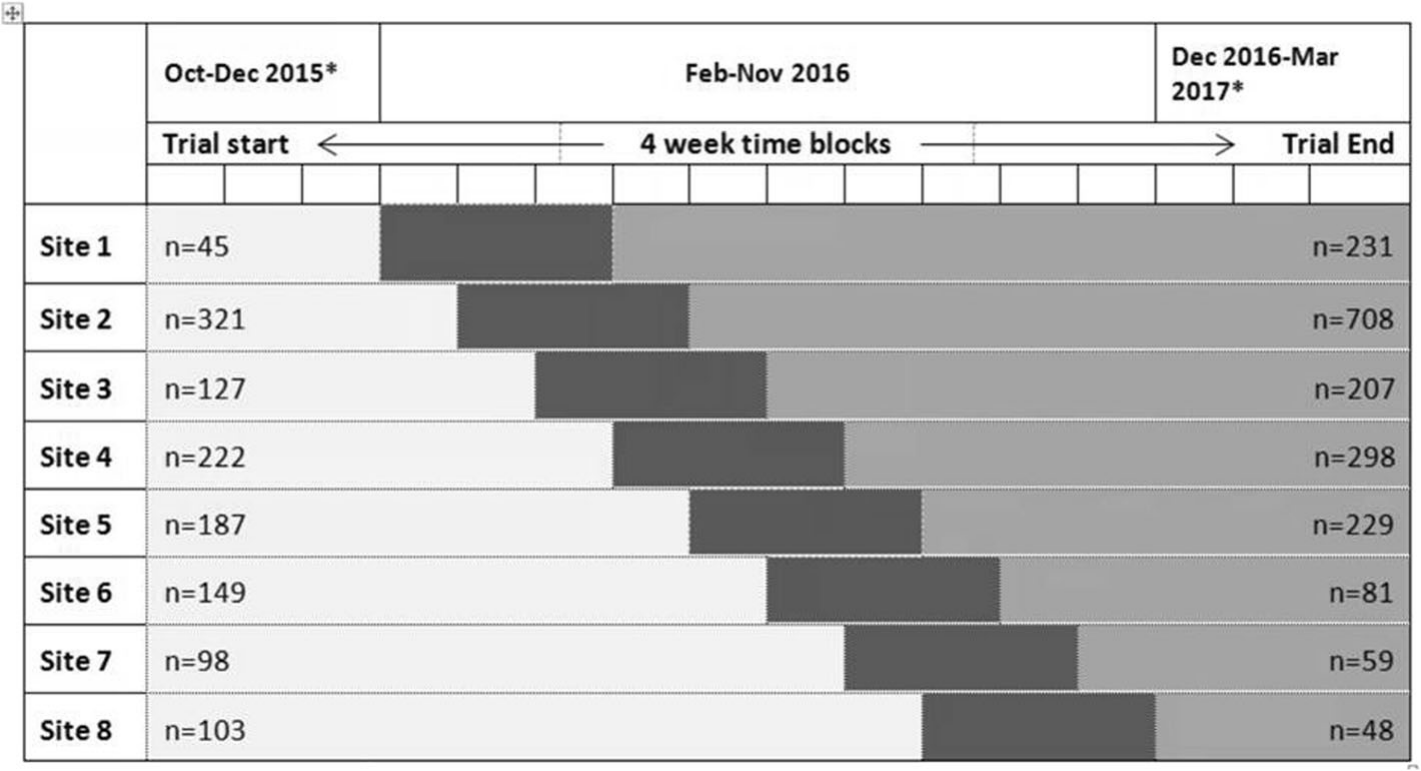
Stepped Wedge trial design. *Excludes period from December 25 to end of January in each year of the trial. Light shade denotes control period, the dark shade the implementation period, and medium shade the intervention period

A stepped wedge cluster randomised controlled trial was conducted [26] in accordance with the published trial protocol [27]. Trial data collection was completed from October 2015 to March 2017. Data were collected from all sites for a 12-week pre-intervention control period. A new site then implemented the intervention every 4 weeks, commencing February 2016. Following the implementation period of 12 weeks at each site, intervention data were collected for a minimum of 12 weeks at all sites (Fig. 1).

The trial was registered with the Australian and New Zealand Clinical Trial Registry (ACTRN12615001016527) [27] and ethical approval was provided by the Human Research Ethics Committee of the health network. Funding was provided by the Australian National Health and Medical Research Council (APP1076777) and the Victorian Department of Health and Human Services.

### Setting

The health network in which the trial took place provides care to a population of more than 700,000 people in eastern metropolitan Melbourne, Australia, and adjacent rural communities. The eight sites within this network that took part in the study met the criteria of providing community outpatient services. For the purposes of the trial, “community outpatient services” were considered to include community health services and sub-acute ambulatory care services (SACS). In Australia, public community health services provide allied health, community nursing services and medical services (within multi-disciplinary teams) to improve health and well-being. They may assist in recovery after an illness or injury, provide support in the management of chronic health conditions, support children with development disabilities or participate in health promotion activities. SACS also offer community-based care, but are usually co-located with other services provided within public health networks [28]. Services are often associated with a hospital admission prevention strategy or follow-up after a hospital stay. For example, they may include specialist, multi-disciplinary teams for assessment and management of conditions such as dementia, incontinence, falls or outpatient multi-disciplinary rehabilitation programs. Sites were selected for inclusion in the trial from 28 community outpatient services within the network that participated in a preliminary study exploring managers’ perceptions of factors that affect waiting lists [9]. Selection for the current trial was based on suitability for the intervention and approval from service managers. Community outpatient services in the network were eligible to participate if they typically provided care over series of appointments, used waiting lists to manage demand and reported the length of their waiting lists to be reasonably stable over the previous 2 years.

### Intervention

All sites used a waiting list to manage demand in the pre-intervention period, with new patients offered appointments as they could be accommodated in clinician schedules. Specific Timely Appointments for Triage (STAT) has been described in detail previously [27]. The fundamental principle of the intervention is that the rate of demand is calculated, and the number of new appointments required each week to keep up with demand is protected in clinician schedules. Patients are allocated an initial appointment immediately after referral (minimising processes associated with access and triage), and prioritisation decisions shift from priority of access to the service to a focus on priority of need for ongoing services after initial assessment. Clinicians make these decisions based on clinical judgement, with access to both a complete picture of client needs and the context of demand for the service.

In line with similar interventions in other settings [21], the intervention began with short-term, targeted interventions involving a small injection of resources that aimed to reduce or clear the backlog of waiting patients. Participating sites were free to use these resources in whatever way they deemed most effective; possibilities included, but were not limited to, employing additional short-term staff, increasing hours of part-time staff or contracting work to private providers. No additional ongoing resources were provided. Team leaders and mangers at each site led the implementation, with project officers from the research team providing education about the intervention and informal support and consultation as required during the implementation period [27].

### Participants

Routinely collected data were analysed from all patients who had their first appointment with the site within the control and intervention periods at each site. New patients were not recruited during the 12-week implementation period, and data collection was also suspended at all sites during the Christmas holiday period because several participating sites either closed or markedly reduced services during this period. Service use of all participating patients was followed for 12 weeks from the date of first appointment.

### Randomisation

The order of intervention for the eight sites was determined through generation of a random sequence using an online randomisation generator (http://www.randomization.com). This was performed in a concealed manner using a single block by a researcher not involved in recruitment or data collection. Service providers were not informed of the order of implementation until eight sites had been recruited and consented to the project.

### Outcome measures

The primary outcome was time in days from referral to first appointment at the level of the patient. Secondary outcome data were collected to determine impacts of the change in model of access on other aspects of service provision at the level of the patient. These were as follows: the rate of missed appointments, the total number of appointments received, the proportion of patients discharged at the end of 12 weeks, the time between the first and second appointment, and the number of unplanned admissions and resulting number of unplanned days spent in hospital in the 6 months following the first appointment with the included site as a marker of adverse events.

Additional variables were collected at both patient and site levels to evaluate the impact of other factors that may have influenced outcomes. These included age, sex, date of referral (reflecting season) and size of the site. The ratio of referrals from the same 12-week time periods over 2 years was also collected for each site as an indicator of any changes in service demand over the course of the project.

Patients attending for the first time during the study period and therefore meeting eligibility for inclusion in the sample were identified prospectively from clinician schedules. Primary and secondary outcome data were then collected from the health network database for each of these patients. Information from databases was supplemented with manual checking of clinician schedules and written referrals to verify accuracy of data or follow up missing information as required.

### Other outcome measures

Health utilisation, cost data, quality of life and service satisfaction data were collected from a sample of 557 patients across the eight sites that contributed to a health economics analysis. In-depth interviews were also conducted with 20 staff members who experienced the change to evaluate the process of implementing the STAT model. Findings of these additional analyses will be reported separately.

### Sample size

A sample of 2496 participants was estimated as the minimum required to detect a mean difference with small to medium effect size in waiting time at 5% level of significance, power of 80% and an intraclass correlation coefficient (ICC) of *ρ* = 0.01 [29, 30]. The calculation estimated approximately 26 admissions per site per 4-week block of data collection. The sample size calculation was based on conservative estimates of the effect size detected in the pilot trial (*δ* = .65) [22] consistent with similar effect sizes observed in studies of Advance Access in general practice settings [31], as well as the number of steps, the number of baseline measurements and the number of measurements between steps in the stepped wedge design.

### Statistical analysis

Waiting time (a count variable) from referral to first scheduled appointment was modelled using generalised mixed effects models assuming a negative binomial dependent variable to allow for over-dispersion. The model was used to assess the effectiveness of the intervention while adjusting for potential confounders such as patient age, patient gender, referral season (summer, autumn, winter and spring), size of the site and demand ratio. A random effect was included for clustering within the site to allow for within-site correlation. Since the intervention effect was likely to vary across clusters, we followed the advice of Davey et al. [32] and ensured that this was adequately modelled. To do so, we introduced a slope random effect for treatment. As a sensitivity analysis, a Gaussian linear mixed effects model was used, allowing for different variances within clusters and both a random intercept and slope were fitted to the log of waiting time plus one. Analyses were completed using the statistical package R version 3.3.3 [33].

## Results

Data were collected from 3113 participants, 1252 in the pre-intervention period and 1861 in the post-intervention period. Characteristics of the sample are included in Table 1. Patient characteristics appeared well matched between control and intervention periods, although differing lengths of time in the control and intervention period for each site due to the trial design contributed to some observed differences. For example, the greater number of patients referred for physiotherapy services and musculoskeletal disorders in the post intervention group was accounted for by site 2 (the largest site) having a short pre and long post intervention period.

**Table 1:** Participant characteristics

	Control period	Intervention period
Patient characteristics	*n* = 1252	*n* = 1861
Gender *[n (%)]*		
Female	743 (59%)	1172 (63%)
Male	509 (41%)	689 (37%)
Age *[years, mean(SD)]*	43 (30)	41 (29)
Referral reason *[n (%)]*		
Musculoskeletal	408 (33%)	862 (46%)
Neurological	113 (9%)	51 (3%)
Developmental assessment	304 (24%)	340 (18%)
Incontinence	350 (28%)	511 (27%)
General function (e.g. falls, mobility, home assessment)	77 (6%)	97 (5%)
Referral source [n (%)]*		
Hospital	243 (19%)	279 (15%)
Medical practitioner	412 (33%)	673 (36%)
Self/relative/carer	146 (12%)	151 (8%)
Community service provider	450 (36%)	757 (41%)
First discipline appointment *n (%)*		
Physiotherapist	695 (56%)	1333 (72%)
Occupational therapist	64 (5%)	70 (4%)
Speech pathologist	179 (14%)	96 (5%)
Nurse	238 (19%)	241 (13%)
Medical specialist	62 (5%)	106 (6%)
Social worker	9 (1%)	6 (< 1%)
Dietician	5 (< 1%)	9 (< 1%)

*One patient with missing data in each group

The eight sites varied both in the nature of the client group (age range, conditions) and service characteristics (size, rural or metropolitan catchment area and staffing mix). Service demand was stable through the period of the trial for five sites; three sites had substantial increases in number of referrals received. Characteristics of included sites are shown in Table 2.

**Table 2:** Characteristics of participating sites

Site characteristics	Number of sites *n* = 8
Classification *(n)*	
Community health service	4
Multidisciplinary SAC clinic	3
Allied Health Outpatient service	1
Service size (clinical EFT) *(median, IQR)*	2.7 (1.5–3.3)
Primary catchment area	
Rural	2
Metropolitan	4
Mixed	2
Disciplines represented *(n)*	
Single-discipline service	3
2–3 disciplines	4
> 3 disciplines	1
Target age group *(n)*	
Paediatric	3
Adult	4
Mixed	1
Primary condition *(n)*	
Continence	2
Neurological	1
Developmental disorders	3
Mixed (ortho/neuro/general frailty)	2
Stability of demand	
No substantial change	5
(< 10% difference, year 1 to year 2)	
25–50% increase	1
50–75% increase	2

Stability of demand was calculated by comparing the number of referrals received from Sept. to Nov. in 2015 (all sites pre intervention) and the same period in 2016 (with each site in either the implementation or post implementation period)

### Implementation of the intervention

The intervention was implemented as planned at each of the eight sites. The mean waiting time of the last 20 patients to be seen at the end of the implementation phase for each site was 33% lower than the mean waiting time for the first 20 patients referred at the start of the implementation phase. This suggests that short-term waitlist reduction strategies (the first component of the intervention) had some effect. A variety of methods, alone or in combination, were used to reduce the numbers of waiting patients during the implementation phase, including extra hours for existing staff (5 sites), additional administrative hours to manage bookings and audit the waitlist (4 sites), employment of additional temporary clinical and/or administrative staff (4 sites) and contracting private service providers (1 site). On average, the investment in waitlist reduction strategies at each site was equivalent to 5% of the annual salary budget (mean AUD$9000) at each site over the 12-week implementation period (range 0.5 to 10%).

### Effect of the intervention: waiting time

**Fig. 2:**
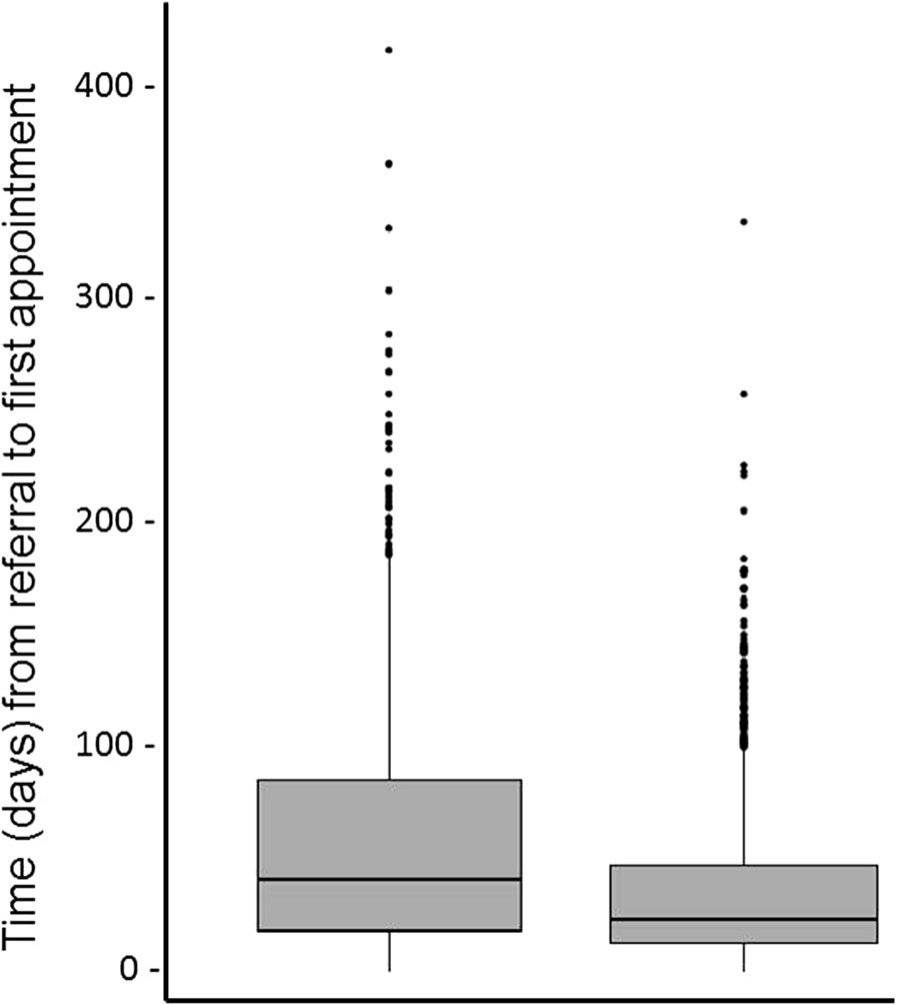
Waiting time for control (left) and intervention (right) periods. Median represented by bar, 25th and 75th percentiles represented by box and upper and lower quartiles represented by whiskers

The intervention resulted in a 33.7% mean reduction in waiting time until first appointment (IRR = 0.663, 95% CI 0.516 to 0.852, *P* = 0.001) (Table 3). Waiting time decreased from a median of 42 days (IQR 19 to 86) in the control period to a median of 24 days (IQR 13 to 48) in the intervention period. The reduction in waiting time during the intervention period was observed to be accompanied by a reduction in variability in waiting time; there appeared to be fewer patients waiting long periods in the intervention period compared with the control period (Fig. 2). Age and gender were significant covariates (age: IRR = 0.997, 95% CI 0.995 to 0.998, *P* < 0.001; gender: IRR = 0.931, 95% CI 0.883 to 0.982, *P* = 0.008), with the mean waiting time estimated to decrease 0.3% per year of increasing age (i.e. older patients were more likely to have shorter waiting times) and, on average, males had an estimated 6.9% lower waiting times than females. Both of these covariates were further tested for interactions with the intervention. Neither interactions were significant suggesting that intervention effects did not significantly differ with respect to gender or age. The findings from the sensitivity analysis were similar in regard to statistical significance and hence not reported further.

**Table 3:** The effect of STAT on time from referral to first appointment (primary outcome)

	Intervention *N* = 1861	Control *N* = 1252	Adj ratio (95% CI)	ICC
Waiting time, *days*				
Mean (SD)	35.6 (33.6)	60.0 (55.2)	IRR 0.663 (0.516 to 0.852)	0.058
Median (IQR)	24 (13–48)	42 (19–86)		

*IRR* incident rate ratio, *ICC* intra-cluster correlation coefficient, *Adj ratio* adjusted ratio indicates that other factors, such as potential confounders, have been included in the model

### Effect of intervention: secondary outcomes

There were no differences in the total number of appointments in the first 12 weeks and the number of days from the first to the second appointment between the intervention and control periods (Table 4). There was little difference in the observed proportion of patients discharged in the first 12 weeks (approximately 50%). However, taking account of clustering, there were reduced odds that patients during the intervention period would be discharged in the first 12 weeks.

**Table 4:** The effect of STAT on secondary outcomes

	Intervention *n* = 1861	Control *n* = 1252	Adj ratio (95% CI)	ICC
Appointments missed per patient, *n*				
*Mean (SD)*	0.5 (0.7)	0.4 (0.9)	IRR 1.18 (1.04 to 1.35)	0.01
*Median (IQR)*	0 (0–1)	0 (0–1)		
Time from the 1st to 2nd appointment, *days, n*				
*Mean (SD)*	28.5 (18.5)	28.8 (18.5)	IRR 1.03 (0.98 to 1.09)	0.03
*Median (IQR)*	23 (13–42)	21 (14–39)		
Appointments in first 12 weeks, *n*				
*Mean (SD)*	2.4 (2.1)	2.1 (1.7)	IRR 0.99 (0.93 to 1.05)	0.01
*Median (IQR)*	2 (1–3)	2 (1–3)		
Patients discharged at 12 weeks, *%*	50.7	48.5	OR: 0.77 (0.60 to 0.99)	0.08
Unplanned admission days, *n*				
*Mean (SD)*	0.4 (4.0)	0.3 (3.5)	IRR 1.33 (0.49 to 3.59)	0.00
*Median (IQR)*	0 (0–0)	0 (0–0)		
Proportion of patients with unplanned hospital admissions within 6 months, *%*	2.3	2.7	OR 1.039 (0.51 to 2.13)	0.24

*IRR* incident rate ratio, *OR* odds ratio, *ICC* intra-cluster correlation coefficient, *Adj ratio* adjusted ratio indicates that other factors, such as potential confounders, have been included in the model

Patients in the intervention period were more likely to miss a scheduled appointment compared to patients in the control period (OR 1.557, 95% CI 1.019 to 2.222). This finding was consistent with observations that the rate of missed appointments increased from 11% in the control period to 16% in the intervention period.

Regarding patient outcomes, there was no difference between the intervention and control periods for the likelihood of having an unplanned hospital admission within 6 months after the first outpatient appointment.

## Discussion

The STAT model (Specific Timely Appointments for Triage) is designed to reduce waiting times for community outpatient services by booking patients directly into protected assessment appointments and combining triage with initial management as an alternative to a waiting list and triage system. A constant rate of patient flow is maintained and calculated to match the rate of referral, and service providers are encouraged to make priority decisions about ongoing treatment in the context of demand. This is the first time that this model has been trialled on a broad scale with multiple services. Findings suggest that the STAT model accounted for a 34% reduction in waiting time after controlling for clustering by site, similar to results of pilot trials conducted in community rehabilitation (42% reduction in waiting time) [22] and physiotherapy outpatients (22% reduction in waiting time) [25]. Thus, this is a feasible way to reduce waiting time across a broad range of community outpatient services, resulting in improved access to care and increased patient flow.

Reductions in waiting time achieved with STAT also appear to be comparable with other patient flow initiatives reported in community outpatient settings, although direct comparison is difficult due to heterogeneity in the ways in which wait times are measured. For example, there was a 23% reduction in median waiting time for a prosthetics clinic through changing scheduling to a modified walk-in system, rather than scheduled appointments [24]; Lynch et al. achieved a 70% reduction in the number of people on a waiting list for mental health services with an intervention that addressed the residual waitlist in combination with new approaches to treatment and triage [23]; and Maddison et al. described a reduction in waiting time for musculoskeletal services despite an increase in referrals through creation of a back pain pathway [34]. In contrast to these studies that all described interventions developed specifically for the services in which they were conducted, the current trial provides evidence of a structured approach that can be used to reduce waiting time across a broad range of settings.

Stability of demand is an important element in the effectiveness and sustainability of the STAT model. Similar to the Advanced Access developed for use in primary care [21], STAT is based partly on the observation that many services have a relatively stable demand, indicated by a waitlist that shows little variation in length over time. Patients are therefore entering the service at a similar rate, but always several weeks or months behind. If the backlog can be cleared and patients brought into a service at a rate that is consistent with the rate of demand, it follows that the service should be able to maintain patient flow without a waiting list developing. However, a sustained increase in demand is one notable risk to the model’s success. This was observed in the current trial as, despite all sites recruited to the trial having reported reasonably stable waiting lists over the previous 2 years, data from two of the sites showed an increase in referrals of more than 50% over the equivalent period in the first compared with the second year of the project, and another site experienced an increase of 25–50%. Despite this, substantial reductions in waiting times were still observed across the eight sites in the trial. This implies that efficiencies driven by STAT may be able to compensate for some increase in demand, but it is likely that a point is reached where additional strategies (such as tightening eligibility criteria or increasing supply) are needed to address the imbalance between supply and demand to achieve ongoing reductions in waiting time. It is also possible that in some services reduced waiting time may stimulate demand, leading to an increase in people seeking the service [1].

In addition to reductions in waiting time, another important outcome was the observed reduction in variability of wait time (Fig. 2). Consistent with a previous pilot study evaluating the STAT model in physiotherapy outpatients [25], the greatest benefits of this intervention appear to have come to those who were previously waiting the longest. This reduction in variability may provide an explanation for how STAT was effective in reducing waiting time overall. This finding is important, as one of the criticisms of traditional waiting lists and triage systems is the risk that low priority patients are continually pushed down the list by those with higher priority ratings, sometimes to the point where they never get seen [8].

The intra-cluster correlation of 0.05 observed for the primary outcome of waiting time was substantially higher than the estimate of 0.01 that was used to determine the sample size in the protocol [27]. This suggests that there was a higher degree of variability between the clusters than originally anticipated and that the impact of the intervention varied to some degree across sites. Given the diversity of sites included in the trial, it is not surprising that there may be site-specific factors that influence the success of the model. The STAT model requires a significant shift in the way that clinicians prioritise their workload, and response to change is likely to have differed to some degree across sites. It is possible also that the STAT model may be more applicable to some settings than others. The planned exploration of the perceptions of key stakeholders at sites where STAT was implemented using qualitative methods will provide insights on the human and service factors that influenced success.

One component of the STAT model is a one-off strategy to reduce the existing backlog prior to implementing the model. A small investment of resources was allocated to each site to facilitate this, and it could be argued that the observed reductions in waiting time were simply a reflection of short-term changes directly related to those additional resources. However, previous literature has shown that single injections of resources, without changes to service delivery, are unlikely to make a sustainable difference to waiting times [35, 36]. STAT is also consistent with other waiting time initiatives that have advocated approaches combining one-off backlog reduction strategies followed by the implementation of patient flow interventions [21, 24]. In the current trial, a comparison of mean waiting times for a small sample of consecutive patients entering the service immediately before and after the waitlist reduction strategies provides some indication of their impact. The 33% reduction in waiting time observed at the conclusion of implementation of targeted waitlist reduction strategies was consistent with the 34% reduction measured across the entire trial. This would suggest that the initial gains made during the backlog reduction strategies were maintained by the STAT model, regardless of the timing of the intervention and relative length of the follow-up period within the stepped wedge design, which continued for up to 10 months.

One perceived risk of an intervention that allows patient flow into a service at a steady rate is the possibility that a “hidden” waitlist is created, where patients receive a first appointment promptly but then wait for a second appointment. The current trial showed no difference in the time from first to second appointment when considering the data across all sites. This finding suggests that concerns about secondary delays were unfounded and that clinicians were prioritising second appointments equally.

There was an increase in the proportion of patients who failed to attend at least one appointment in the intervention period, which was surprising given that failure to attend rates have previously been negatively associated with waiting time [13]. A possible explanation is that patients in the intervention group were given information about their appointment time soon after referral rather than being placed on a waiting list for an interim period. Although overall waiting time reduced during the intervention period, the time between being given an appointment and the appointment itself increased. For example, where previously a patient might wait 6 weeks to receive notification of an appointment 1 week later, with STAT this same patient receives notification after 1 week for an appointment 3 weeks later. Forgotten appointments become more likely and could be mitigated by strategies such as SMS reminders [37].

This trial was conducted in eight community outpatient sites that differed from each other in a number of ways. They provided a range of services to patients ranging from infants to the frail elderly, some treated chronic conditions and others provided short-term follow-up to acute injuries. All sites, however, shared the common features of providing non-emergency services to patients over a series of outpatient appointments. These observations suggest that the STAT model is likely to be generalisable to a wide range of outpatient services provided that they have these features. STAT encourages clinicians to change the focus of decisions about patient priority; rather than triage decisions influencing access to the service, prioritisation is instead directed at the rationing of resources for ongoing treatment. In order for this to work, there needs to be some flexibility in the way that services are delivered. For example, clinicians working in these types of services can choose to see patients less frequently, for shorter appointments, or move patients from individual to group sessions during times of high demand. Results of this trial suggest that STAT is likely to be applicable to any non-emergency outpatient service with stable demand and flexibility in service delivery decisions, regardless of the type of service provided.

A major strength of this trial is the use of a stepped wedge cluster randomised controlled trial design, an emerging trial design in health service delivery evaluations that offers several benefits over other parallel cluster designs [26, 38]. The rigour of this method and the involvement of multiple sites offers clear advantages over other commonly used methods for evaluation of patient flow or waiting list interventions, such as single-site studies [23, 39], quality improvement methods [40, 41] or retrospective analyses of health service datasets [42, 43]. The ICC for the primary outcome of waiting time was larger than hypothesised in our sample size estimation (*ρ* = 0.058 versus *ρ* = 0.01) [27], possibly due to greater variability than expected between sites. There were many possible sources of variation, including differences between clinicians, management of each of the sites, socioeconomic characteristics of the patient population and complexity of patient needs that were not measured in the trial. Despite this, all sites provided services for patients living in the community and the analysis took account of clustering. Further adequately powered studies could investigate the effect of variation, in particular the effect of STAT on subgroups of patients, such as those from lower socioeconomic backgrounds and those with more complex health needs [44, 45].

The stepped wedge design of this trial meant that the lengths of control and intervention data collection varied between sites. As a result, there were some differences in the characteristics of patients in the pre and post intervention data driven by differences between the services, but this was accounted for by clustering in the analysis. This aspect of the design also meant that the last site to receive the intervention had a follow-up period of only 3 months. It is possible that this was not long enough to measure the true effect of the intervention. Conversely, over a longer follow-up period, sustainability of the intervention may come into question, as the effect of the initial injection of resources wears off and support from the research team is withdrawn. The relatively short follow-up time (particularly for the last site to receive the intervention) is a limitation of the current trial, and further research is required to look at longer-term outcomes. A further challenge of the trial design was that it provided for little flexibility in the timing of implementation of the intervention, reducing backlogs and embedding new processes into practice. It is therefore likely that greater benefits may be achieved when implementing STAT without these limitations.

There were some minor deviations from the protocol due to the characteristics of the services selected for inclusion and availability of the required data. We intended to collect data on the number of patients on the waiting list at key time points for each service to assess the fidelity of the implementation strategies to reduce backlog at each site. It was not possible to collect these data across all sites due to differences in the way that waiting list data were recorded. Instead, we analysed waiting time for a sample of 20 consecutive patients at each time point rather than counting the number of patients on the waiting list. We also intended to analyse the number of group and individual appointments across sites and time periods to see whether the new model of care led to increased use of group appointments, as observed in a previous trial [22]; however, this was not necessary as the majority of the sites selected for inclusion in the trial did not offer group appointments as a treatment option.

## Conclusion

A model of access and triage based on evidence-based strategies known to improve patient flow was successfully implemented on a broad scale, involving eight community outpatient services. Waiting time was reduced by 34%, and waiting time variability also decreased substantially, suggesting those people previously waiting the longest were likely to benefit most. This trial also demonstrated that evaluation of patient flow initiatives previously limited to single-site studies, quality improvement projects or retrospective analysis of health service data can be conducted using rigorous research methods to produce high-quality evidence for health care service providers.

## Abbreviations

SACS: Sub-acute ambulatory care services
STAT: Specific Timely Appointments for Triage
